# Associations of bullying victimisation in different frequencies and types with suicidal behaviours among school-going adolescents in low- and middle-income countries

**DOI:** 10.1017/S2045796022000440

**Published:** 2022-08-11

**Authors:** Wenjing Fei, Shun Tian, Hongshu Xiang, Yiran Geng, Jiachun Yu, Chen-Wei Pan, Tianyang Zhang

**Affiliations:** 1 Jiangsu Key Laboratory of Preventive and Translational Medicine for Geriatric Diseases, School of Public Health, Medical College of Soochow University, Suzhou, China; 2 Research Center for Psychology and Behavioral Sciences, Soochow University, Suzhou, China

**Keywords:** Adolescents, bullying victimization, global mental health, suicidal behaviours

## Abstract

**Aims:**

Adolescent suicide is a severe public health problem in low- and middle-income countries (LMICs), and adolescents who are victims of bullying have a higher risk of suicidal behaviours. However, detailed global data concerning the association between bullying victimisation and suicide are lacking; thus, further multicontinental studies exploring the association of bullying victimisation at different frequencies and types with suicidal behaviours are urgent.

**Methods:**

The data were extracted from the Global School-based Student Health Survey (GSHS) (2010–2017) conducted in 40 LMICs (*n* = 151 184, mean age: 14.77 years, s.d.: 1.59, 54.2% females). Data concerning past-30-day bullying victimisation, past 12-month suicidal behaviours (suicidal ideation, suicidal plans and suicidal attempts) and other adverse health behaviours or outcomes were collected. Chi-square tests were used to explore the correlations among the main variables. A multivariable logistic regression and stratified logistic regressions were conducted to assess the associations.

**Results:**

The overall prevalence of bullying victimisation, suicidal ideation, suicidal plans and suicidal attempts were 28.72, 12.64, 11.84 and 10.79%, respectively. The results showed a positive association of different frequencies and types of bullying victimisation with suicidal behaviours: suicidal ideation (odds ratio (OR) = 2.43, 2.06–2.87), suicidal plans (OR = 2.69, 2.28–3.17) and suicidal attempts (OR = 3.23, 2.73–3.82). Adolescents also reported the effects of being made fun of because of their religion: suicidal ideation (OR = 1.63, 1.41–1.88), suicidal plans (OR = 1.44, 1.24–1.66) and suicidal attempts (OR = 1.73, 1.50–1.98). Moreover, these associations varied among teenagers of different gender and body mass indexes (BMIs) and were stronger among males and adolescents who were underweight, overweight or obese.

**Conclusions:**

Different types of bullying victimisation were positively related to suicidal behaviours; these associations varied among adolescents by gender and BMI. This study offers a theoretical basis for the identification of adolescents at a high risk of suicide and is beneficial for informing effective psychological interventions for constructing sound school environments, improving adolescents’ mental health and reducing the risk of suicide to promote health in LMICs and globally.

## Introduction

In 2019, suicide was the second leading cause of death among adolescents and young adults aged 10–24 years (WHO, 2019). Suicidal ideation tends to emerge during early adolescence (10–13 years) (Geoffroy *et al*., 2021). Importantly, low- and middle-income countries (LMICs) account for more than 79% of all suicides worldwide, and they are home to the vast majority (90%) of youth (Department of Economic and Social Affairs, 2019). Thus, there is an urgent need to identify potentially modifiable risk factors. In particular, bullying victimisation has gained increasing attention (Arango et al., 2016; Moore *et al*., 2017; Klomek *et al*., 2019; Koyanagi *et al*., 2019).

### Suicidal behaviours

Suicidal behaviours can result in significant medical, social and economic burdens and have profound psychological effects on the individual and families (Koyanagi *et al*., 2019). The three stages before completed suicide, including suicidal ideation, suicidal plans and suicidal attempts, deserve more attention. Adolescence is a vulnerable stage in one's life when one is exposed to heightened risks and challenges. Suicidal behaviours develop during adolescence and peak during late adolescence and early adulthood (Goldman-Mellor *et al*., 2014; Mars *et al*., 2014; Finkelstein *et al*., 2015); thus, it is critical to identify risk factors for suicidal behaviours in adolescence.

Suicidal behaviours are complex phenomena that are rooted in nonlinear interactions with multiple risk indicators (Goldman-Mellor *et al*., 2014; Twenge *et al*., 2018; Liu *et al*., 2020). Rates of nonfatal suicidal behaviours differ across adolescents. For example, the prevalence of suicidal ideation, suicidal plans and suicidal attempts is higher among females (Nock *et al*., 2013), while the prevalence of completed suicides is higher among men and older people worldwide (WHO, 2021). Adolescents who are overweight or obese (Klinitzke *et al*., 2013) and display delinquent behaviours or lack social support are also high-risk groups for suicidal behaviours (Su *et al*., 2019; Rahman *et al*., 2020). However, suicide has been found to be prevalent among adolescents involved in bullying, which has higher incidence in LMICs. Therefore, bullying victimisation can be considered a significant factor in suicidal behaviours among adolescents in LMICs.

### Bullying victimisation

A student can be involved in bullying as a victim, a bully or a bully-victim (Mark, 2019). Several previous studies have shown greater risk for mental health problems among victims of bullying (Wolke *et al*., 2013; Eisenberg *et al*., 2015). Bullying victimisation in adolescence is highly prevalent in some LMICs (Koyanagi *et al*., 2019). In addition, the rate of victimisation varies and generally declines with age. As children age, physical bullying tends to decline, while verbal and relational bullying increases. Boys are more likely to be bullied physically, while girls are more often bullied verbally (Craig *et al*., 2009). Additionally, adolescents who are underweight, overweight or obese have significantly greater odds than their healthy-weight peers of being the victims of bullying (Rupp and McCoy, 2019).

### Association of bullying victimisation and suicidal behaviour

The associations between bullying victimisation and suicidal behaviours have been well established in the past two decades (Espelage and Holt, 2013; Holt *et al*., 2015; Arango et al., 2016; Moore *et al*., 2017; Klomek *et al*., 2019). A systematic review focusing on the link between bullying victimisation and suicidality in the general population of youth found odds ratios (ORs) ranging from 1.4 to 10.0 in cross-sectional studies and from 1.7 to 11.8 in longitudinal studies (Klomek *et al*., 2010).

The association between bullying victimisation and suicidal behaviours has not always been the same across different cases. Some studies have identified social protective factors against suicidal behaviour among victims of bullying. Moreover, findings with respect to gender differences are mixed (Laukkanen *et al*., 2005; Klomek *et al*., 2009), which can be the focus of our study. Considering that body mass index (BMI) has a significant relationship with bullying victimisation and suicidal behaviour, we supposed that BMI is a moderating factor in bullying and suicidal behaviours.

### The present study

Previous studies have mainly focused on the associations between bullying victimisation and suicidal ideation or suicidal attempts among adolescents. However, our research focused on the three components of suicide and was a multicontinental study covering 40 LMICs and examined both the frequency and the different types of bullying. In addition, we also considered some factors as moderators in the association between bullying victimisations and suicidal behaviours. Thus, there were multiple hypotheses of the present study.

Hypothesis 1. The prevalence of bullying victimisations and suicidal behaviours varies in different adolescents.

Hypothesis 2. Bullying victimisation is positively associated with suicidal behaviours, and this applies to different types of bullying.

Hypothesis 3. Gender and BMI moderate the relation between bullying victimisation and suicidal behaviours.

## Methods

### The survey

The Global School-based Student Health Survey (GSHS) initiated by the WHO uses a self-administered questionnaire to obtain data concerning health behaviours and protective factors related to disease and death among students. The details of this survey can be found at http://www.who.int/chp/gshs and http://www.cdc.gov/gshs.

We excluded survey responses collected before 2009 because our study aimed to enhance the comparability of findings between countries, and outdated records would inevitably impact a justifiable evaluation. For countries that completed more than one GSHS, we analysed only the most recent survey. Some additional information on this survey is available in online Supplementary material 1.

### Bullying victimisation

In the GSHS questionnaire, the definition of bullying (a student or group of students saying or doing bad and unpleasant things, teasing another student in an unpleasant way or leaving another student out of things on purpose) was provided to students before they answered the questions. Bullying victimisation was assessed in two parts. First, the frequency of bullying victimisation was assessed by the following question: ‘During the past 30 days, on how many days were you bullied?’ The options were ‘0 days’, ‘1–2 days’, ‘3–5 days’, ‘6–9 days’, ‘10–19 days’, ‘20–29 days’ and ‘all 30 days’. Then, the different types of bullying were assessed by the following single-choice question: ‘During the past 30 days, how were you bullied most often?’ The options were ‘I was not bullied during the past 30 days’, ‘I was hit, kicked, pushed, shoved around or locked indoors’, ‘I was made fun of because of my race, nationality or colour’, ‘I was made fun of because of my religion’, ‘I was made fun of with sexual jokes, comments or gestures’, ‘I was left out of activities on purpose or completely ignored on purpose or completely ignored’, ‘I was made fun of because of how my body or face looks’ or ‘I was bullied in some other way’. According to the forms of bullying victimisation, the first is classified as physical (physical threats and harm), the fifth is considered relational (excluding and spreading rumours) and the other types are considered verbal (teasing and calling names) (Smith *et al*., 2002).

### Suicidal behaviours

Suicide is death caused by self-directed injurious behaviour with the intent to die as a result of the behaviour, which has the following four components that can be considered a continuum: suicidal ideation, suicidal plans, suicidal attempts and consummate suicide (American Psychiatric Association, 2013; WHO, 2014; Dong *et al*., 2018; Jans *et al*., 2018). In the present study, suicidal behaviours included suicidal ideation, suicidal plans and suicidal attempts. Suicidal ideation was assessed by the following question: ‘During the past 12 months, did you ever seriously consider attempting suicide?’ The options for a response were ‘Yes’ or ‘No’. Suicidal plans were assessed by the following question: ‘During the past 12 months, did you make a plan about how you would attempt suicide?’ The options for a response were ‘Yes’ or ‘No’. Suicidal attempts were assessed by the following question: ‘During the past 12 months, how many times did you actually attempt suicide?’ The options for a response were ‘0 times’, ‘1 time’, ‘2 or 3 times’, ‘4 or 5 times’ and ‘6 or more times’. In this study, we coded the answer ‘0 times’ as ‘does not have experience with suicidal attempts’ and the responses ‘1 time’, ‘2 or 3 times’, ‘4 or 5 times’ and ‘6 or more times’ as ‘has experience with suicidal attempts’.

### Control variables

Age, gender, BMI, area, food insecurity, delinquent conduct (cigarette smoking, alcohol use, marijuana use), other mental health problems (loneliness and sleeping difficulty) and experiences at home and school (missing school, having parental understanding, having close friends) were included as covariates.

### Statistical analyses

The statistical analyses were conducted in SPSS 21.0 and SAS 9.4. Descriptive analysis was performed. Rao–Scott and Pearson chi-square tests were used to explore the correlations between the main variables. The Bonferroni method was performed for pairwise comparisons between two groups. Multinomial logistic regressions were conducted to examine the associations between bullying victimisation and suicidal behaviours, which included covariates. Furthermore, stratified logistic regressions were conducted according to participants' gender and BMI.

## Results

### Demographic characteristics of the participants

We identified 40 LMICs with the GSHS datasets. A total of 151 184 participants (mean age: 14.77 years old, s.d.: 1.59) were included after the cases with missing values were removed. The detailed distribution of the demographic characteristics of the participants is shown in Table 1 and Table S1 in online Supplementary material 3.

**Table 1.** Sample characteristics (*n* = 151 184)

	*n*	%
Age		
11–13 years old	35 763	23.66
14 years old	32 857	21.73
15 years old	32 730	21.65
16 years old	26 940	17.82
17–18 years old or older	22 894	15.14
Gender		
Male	69 299	45.84
Female	81 885	54.16
BMI		
<18.5	41 928	27.73
18.5–24.9	92 455	61.15
25–30	11 484	7.60
>30	5317	3.52
Frequency of bullying victimisation		
0 days	107 764	71.28
1 or 2 days	27 098	17.92
3–5 days	7453	4.93
6–9 days	3019	2.00
10–19 days	1906	1.26
20–29 days	895	0.59
All 30 days	3049	2.02
Different types of bullying		
Not bullied	116 785	77.25
Type 1	4671	3.09
Type 2	3721	2.46
Type 3	1415	0.94
Type 4	5727	3.79
Type 5	2115	1.40
Type 6	5901	3.90
Type 7	10 849	7.18
Suicidal ideation		
No	132 067	87.36
Yes	19 117	12.64
Suicidal plans		
No	133 290	88.16
Yes	17 894	11.84
Suicidal attempts		
No	134 877	89.21
Yes	16 307	10.79

*Note*: Type 1: kicked, pushed or shoved around or locked indoors; type 2: made fun of race, nationality or colour; type 3: made fun because of religion; type 4: made fun of with sexual jokes, comments or gestures; type 5: left out of activities on purpose or completely ignored; type 6: made fun of about body or face looks; type 7: some other way. (Additional information about sample characteristics is provided in Table S1 of online Supplementary material 3.)

### Prevalence of bullying victimisation, suicidal ideation, suicidal plans and suicidal attempts

Table 1 shows that 28.72% of students reported being victimised for at least 1 day. The prevalence of bullying victimisation at a higher frequency was lower. The prevalence of different types of bullying is shown in the table. In addition, the rates of suicidal ideation, suicidal plans and suicidal attempts were 12.64, 11.84 and 10.79%, respectively.

### Differences in bullying victimisation and suicidal behaviours

Table 2 shows the difference in bullying victimisation during the past 30 days in several aspects. Most of the pairwise comparisons between the two groups were significantly different (*p* < 0.05). Taking age as an example, the students aged 11–13 years had the highest prevalence of bullying victimisation (24.82%). Being made fun of because of how one's body or face looks had the highest prevalence among the students aged 11–16 years. In students aged 17–18 years old or older, being made fun of with sexual jokes, comments or gestures (3.43%) had the highest prevalence. Moreover, the prevalence of bullying victimisation (by BMI, area and other control variables) is shown in Table 2 and Table S2 in online Supplementary material 3. A detailed description of this table and the results of the interaction analysis are provided in online Supplementary material 2.

**Table 2.** Factors associated with bullying victimisations among adolescents (*n* = 151 184)

	Different types of bullying (%)								Rao–Scott chi-square *p*
	Not bullied	Type 1	Type 2	Type 3	Type 4	Type 5	Type 6	Type 7	
Age									<0.001
11–13 years old	75.18	3.97	2.73	0.86	3.74	1.54	3.99	7.99	
14 years old	76.09	3.25	2.48	0.93	4.16	1.40	4.32	7.37	
15 years old	77.42	2.82	2.41	0.90	3.69	1.46	4.08	7.22	
16 years old	78.79	2.44	2.31	0.91	3.83	1.26	3.85	6.61	
17–18 years old or older	80.06	2.64	2.27	1.15	3.43	1.25	2.97	6.22	
Gender									<0.001
Male	76.40	4.12	2.78	1.00	4.15	1.22	3.17	7.14	
Female	77.96	2.21	2.19	0.88	3.48	1.55	4.52	7.21	
BMI									<0.001
<18.5	76.40	3.37	2.62	0.88	4.37	1.48	3.54	7.35	
18.5–24.9	77.42	3.08	2.47	0.99	3.65	1.45	3.61	7.34	
25–30	78.65	2.63	2.00	0.86	3.32	0.95	5.63	5.96	
>30	77.90	2.07	2.07	0.68	2.63	0.94	8.11	5.60	
Areas									<0.001
African	69.59	5.75	4.08	2.26	2.75	1.66	4.34	9.57	
Americas	79.39	1.69	1.55	0.54	2.86	1.38	4.87	7.70	
Eastern Mediterranean	89.32	2.17	0.98	0.58	1.43	0.34	1.03	4.16	
South-East Asia	76.22	3.02	1.62	0.87	4.56	1.31	2.93	9.47	
Western Pacific	77.65	3.25	2.95	0.85	4.46	1.44	3.79	5.60	

*Note*: Type 1: kicked, pushed or shoved around or locked indoors; type 2: made fun of race, nationality or colour; type 3: made fun because of religion; type 4: made fun of with sexual jokes, comments or gestures; type 5: left out of activities on purpose or completely ignored; type 6: Made fun of about body or face looks; type 7: some other way. (Additional information is provided in Table S2 of online Supplementary material 3.)

In addition, Table 3 shows the difference in suicidal behaviours in the same aspects. Most of the pairwise comparisons between the two groups were significantly different (*p* < 0.05). Taking age as an example, the prevalence of suicidal behaviours declined with increasing age. Students aged 11–13 years had the lowest prevalence of suicidal ideation (10.64%), suicidal plans (9.95%) and suicidal attempts (9.09%), while students aged 17–18 years or older had the highest prevalence, i.e. 13.12, 13.21 and 11.2%, respectively. In addition, the prevalence of suicidal behaviours (by BMI, area and other control variables) is shown in Table 3 and Table S3 in online Supplementary material 3. A detailed description of this table is provided in online Supplementary material 2.

**Table 3.** Factors associated with suicidal behaviours among adolescents (*n* = 151 184)

	Suicidal ideation		Suicidal plans		Suicidal attempts	
	Prevalence (%)	*p*	Prevalence (%)	*p*	Prevalence (%)	*p*
Age		<0.001		<0.001		<0.001
11–13 years old	10.64		9.95		9.09	
14 years old	12.61		11.73		11.09	
15 years old	13.57		12.45		11.55	
16 years old	13.82		12.56		11.39	
17–18 years old or older	13.12		13.21		11.20	
Gender		<0.001		<0.001		<0.001
Male	9.84		9.86		9.17	
Female	15.01		13.51		12.15	
BMI		<0.001		<0.001		<0.001
<18.5	10.11		9.46		8.85	
18.5–24.9	13.56		12.69		11.67	
25–30	14.14		13.20		11.02	
>30	13.43		12.77		10.14	
Areas		<0.001		<0.001		<0.001
African	16.48		18.18		16.55	
Americas	17.43		15.30		13.90	
Eastern Mediterranean	12.33		8.13		8.02	
South-East Asia	7.72		7.84		5.18	
Western Pacific	10.77		9.93		9.75	
Food insecurity		<0.001		<0.001		<0.001
No	10.68		10.03		8.53	
Yes	14.53		13.56		12.94	
Smoking cigarette		<0.001		<0.001		<0.001
No	11.06		10.37		9.21	
Yes	24.48		22.77		22.59	
Alcohol use		<0.001		<0.001		<0.001
No	10.20		9.54		8.42	
Yes	21.58		20.22		19.41	
Marijuana use		<0.001		<0.001		<0.001
No	11.97		11.18		9.99	
Yes	31.79		30.44		33.55	
Missed school		<0.001		<0.001		<0.001
No	10.95		10.26		8.75	
Yes	17.00		15.89		16.01	
Parental understanding		<0.001		<0.001		<0.001
No	18.38		16.79		14.52	
Yes	11.02		10.43		9.73	

Additional information is provided in Table S3 of online Supplementary material 3.

### Associations between bullying victimisation and suicidal behaviours

**Fig. 1.**
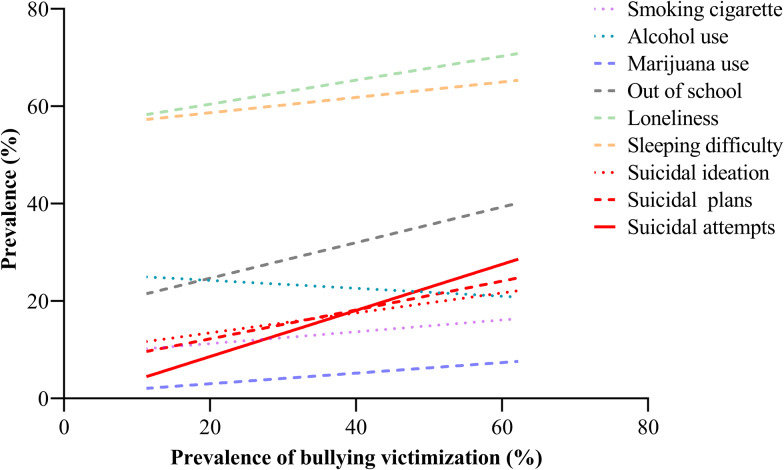
Associations of bullying victimisation with suicidal behaviours and other behavioural or emotional problems (*n* = 151 184).

Figure 1 shows that as the frequency of bullying victimisation increased, the prevalence of suicidal behaviours and other behavioural or emotional problems among the students increased.

In the multinomial logistic regression models (Table 4), the associations between bullying victimisation and suicidal behaviour were significant after adjusting for control variables. Taking different frequencies of bullying victimisation as an example, using 0 days of bullying victimisation as a reference, the risk of suicidal ideation at 20–29 days and all 30 days (OR = 2.31, 95% confidence interval (CI) = 2.07–2.56) was higher. The odds of suicidal plans and suicidal attempts also increased as the frequency of bullying victimisation increased; the details are shown in the table. Other details are shown in Table 4, and a detailed description of this table is provided in online Supplementary material 2.

**Table 4.** Logistic regression results (*n* = 151 184)

	Suicidal ideation		Suicidal plans		Suicidal attempts	
	OR (95% CI)	*p*	OR (95% CI)	*p*	OR (95% CI)	*p*
Frequency of bullying victimisation						
0 days						
1 or 2 days	1.30 (1.22–1.39)	<0.001	1.34 (1.25–1.43)	<0.001	1.80 (1.68–1.92)	<0.001
3–5 days	1.53 (1.40–1.67)	<0.001	1.61 (1.47–1.75)	<0.001	2.46 (2.26–2.68)	<0.001
6–9 days	1.83 (1.64–2.04)	<0.001	1.87 (1.68–2.09)	<0.001	2.55 (2.28–2.85)	<0.001
10–19 days	2.07 (1.82–2.35)	<0.001	2.03 (1.78–2.31)	<0.001	2.60 (2.28–2.96)	<0.001
20–29 days	2.43 (2.06–2.87)	<0.001	2.69 (2.28–3.17)	<0.001	3.23 (2.73–3.82)	<0.001
All 30 days	2.31 (2.07–2.56)	<0.001	2.25 (2.02–2.50)	<0.001	3.11 (2.79–3.46)	<0.001
Different types of bullying						
Not bullied						
Type 1	1.47 (1.33–1.62)	<0.001	1.44 (1.31–1.59)	<0.001	1.41 (1.29–1.55)	<0.001
Type 2	1.34 (1.21–1.49)	<0.001	1.30 (1.17–1.45)	<0.001	1.32 (1.19–1.46)	<0.001
Type 3	1.63 (1.41–1.88)	<0.001	1.44 (1.24–1.66)	<0.05	1.73 (1.50–1.98)	<0.001
Type 4	1.46 (1.33–1.60)	<0.001	1.31 (1.19–1.44)	<0.01	1.26 (1.15–1.38)	<0.001
Type 5	1.48 (1.31–1.68)	<0.01	1.27 (1.12–1.45)	<0.01	1.11 (0.98–1.26)	>0.05
Type 6	1.45 (1.32–1.58)	<0.001	1.28 (1.17–1.41)	<0.01	0.98 (0.90–1.08)	>0.05
Type 7	1.19 (1.10–1.29)	<0.05	1.11 (1.03–1.21)	<0.05	0.92 (0.85–1.00)	>0.05

*Note*: Type 1: kicked, pushed or shoved around or locked indoors; type 2: made fun of race, nationality or colour; type 3: made fun because of religion; type 4: made fun of with sexual jokes, comments or gestures; type 5: left out of activities on purpose or completely ignored; type 6: made fun of about body or face looks; type 7: some other way.

**Fig. 2.**
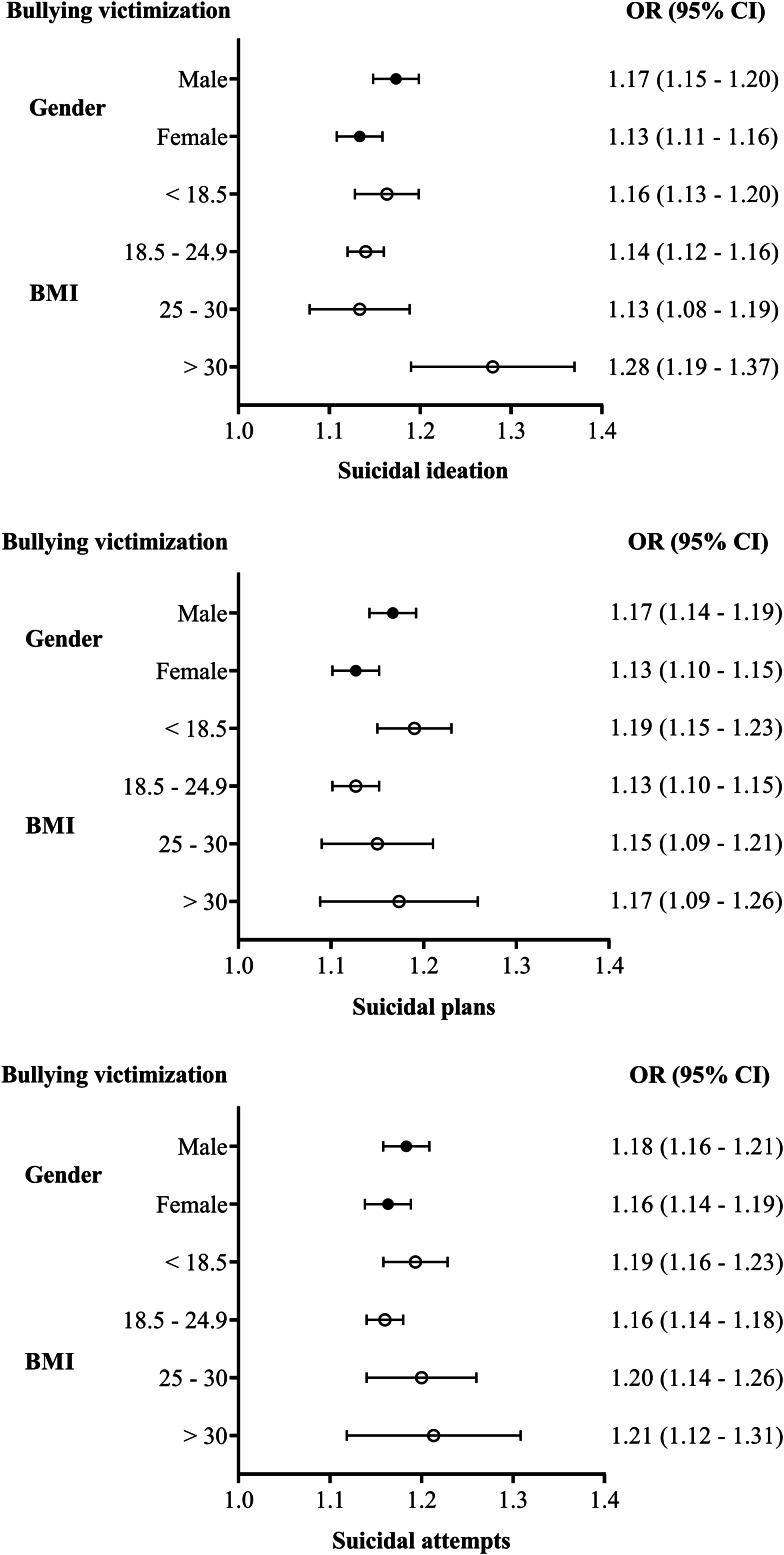
Association between bullying victimisation and suicidal behaviours estimated by stratified logistic regressions (*n* = 151 184). *Note*: OR, odds ratio; CI, confidence interval.

Furthermore, the results of stratified logistic regressions conducted according to participants' gender and BMI are shown in Fig. 2. Taking gender as an example, compared to females, males experiencing bullying victimisation had a higher risk of suicidal ideation (OR = 1.17, 95% CI = 1.15–1.20), suicidal plans (OR = 1.17, 95% CI = 1.14–1.19) and suicidal attempts (OR = 1.18, 95% CI = 1.16–1.21). The other details are shown in the figure, and a detailed description is provided in online Supplementary material 2.

## Discussion

The high prevalence rates reported in this study confirm that bullying and suicidal behaviours among adolescent students are alarmingly commonplace in impoverished nations, thus underscoring the need for preventive intervention research targeting bullying victimisation in schools to better protect adolescents from such adverse experiences during this crucial period of their lives.

### Bullying victimisation

Similar to previous studies (Barzilay *et al*., 2017), verbal and physical victimisation occurred at fairly high levels, while relational bullying was relatively low, and the prevalence varied in different situations, which was consistent with hypothesis 1. The frequency of victimisation decreased with the age of adolescents. This reduction by age could be attributable to age-related changes in youth adapting socially as they develop or reflect equalisation in physical sizes and consequently (Craig *et al*., 2009; Tan *et al*., 2019). Regarding the types of bullying, the risk of being made fun of in sexual forms was higher among older adolescents in African, American and Eastern Mediterranean countries, while younger adolescents from the Western Pacific region also had a higher risk of this type of bullying. Moreover, the risk of being made fun of about one's body was higher among older adolescents from Africa and younger adolescents from Southeast Asia. The incidence of bullying victimisation in adolescents with food insecurity in Africa was relatively high, and physical forms were the most common. This finding may be due to differences in culture, economic development and educational systems across the areas, leading to inconsistent levels of adolescents' physical and psychological development. Moreover, consistent with Chinese research (Fung *et al*., 2021), bullying victimisation was more popular among male adolescents, who had higher prevalence of the physical forms, while females had higher prevalence of being made fun of in sexual forms. One possible explanation is that the stereotypical participation of boys and girls in situations of bullying has social roots; more aggressive behaviours and violence are reinforced among boys, whereas the victimisation of girls is more consistent with traditional stereotypes of femininity (Marta *et al*., 2013). Furthermore, sexual maturity occurs earlier in females, resulting in a vulnerability to victimisation peaking during the initial states of sexual development (Fernández *et al*., 2013). In addition, teenagers with the lowest BMI seemed to lack self-protection ability and had the highest prevalence of bullying victimisation. Obese adolescents were more susceptible to being made fun of how their body or face looked, which is consistent with a previous study. Adolescents who were underweight and overweight or obese had significantly greater odds than their healthy-weight peers of being victims of bullying (Rupp and McCoy, 2019).

Furthermore, this study found strong associations between bullying victimisation and adverse health behaviours and other mental disorders in adolescents, which lends further support to research findings from other scholars (Ndibalema, 2013). One possible explanation could be that the experience of bullying during vulnerable developmental adolescent periods leads to neurobiological (Shonkoff *et al*., 2009; Li *et al*., 2021) and inflammatory (Copeland *et al*., 2014) changes that then cause mental illness (Shonkoff *et al*., 2009); then, they attempt to self-medicate their distress and negative emotions with tobacco or alcohol, resulting in increased problems with parents and the school atmosphere.

### Suicidal behaviours

The prevalence of suicidal behaviours varied in different situations, which was consistent with hypothesis 1. Younger participants were less likely to have suicidal behaviours, which may partly be because they shoulder fewer responsibilities. The study performed by David *et al*. (2001) supported our results. The rates of suicidal behaviours in females were higher. Psychological autopsy studies have revealed that many women have low sociocultural status and are sensitive or show emotional fragility (Rosenfield and Smith, 2010). Consistent with previous research (Klinitzke *et al*., 2013), adolescents who are overweight or obese had a higher rate of suicidal behaviours, which may due to their severe physical or psychological burden. Moreover, the rate of suicidal behaviours among teenagers in Southeast Asia was significantly lower, while the highest incidence was in African countries, where information on suicidal behaviours and mental health is particularly lacking. Research and public health efforts are mainly directed towards infectious diseases such as tuberculosis, malaria and AIDS (Uchechukwu *et al*., 2019; Gioseffi and Brignol, 2020). Thus, the socioeconomic and cultural context for suicide in different areas should be mentioned, and the limited availability or low utilisation of mental health systems could contribute to the reduced detection of mental disorders that can lead to suicide (Knipe *et al*., 2017).

In addition, our results identify some factors associated with suicidal behaviours. Prior studies also suggested that psychosocial distress and risky health behaviours were associated with suicidal ideation (Page and West, 2011; Perez *et al*., 2016). Notably, bullying victimisation might also contribute to suicidal behaviours through accumulative internalised behaviours, such as social isolation, shame and feelings of depression, which eventually affect individuals' ability to deal with stressors associated with bullying victimisation (Page and West, 2011). Effective bullying prevention programmes and continued large-scale longitudinal research that focuses on the short- and long-term implications of adolescents' bullying victimisation and its possible linkage should be the areas of further research. The association between bullying victimisation and suicidal behaviours may be mediated by these factors.

### The association between bullying victimisation and suicidal behaviours

Bullying victimisation was an independent risk factor for suicidal behaviours among adolescents in LMICs, which was in line with hypothesis 2 and previous cross-sectional and longitudinal studies (Messias *et al*., 2014; Beop-Rae *et al*., 2015; Koyanagi *et al*., 2019), providing converging evidence that this association may truly be a global phenomenon. A greater number of days bullied was associated with increasing odds of suicidal behaviours, which is in line with emotional regulation theory (Gross, 2015). Individuals who have had bad experiences are more likely to have psychological maladjustment, and such continuous accumulation of bad experiences will lead to suicide. Regarding the different types of bullying, being made fun of because of religion had the strongest predictive effect on suicidal behaviours. This phenomenon may be related to identity cognition (such as religion and race). Religious identity often plays an important role in adolescents' development of self-identity. Identity-based bullying may lead minorities to experience greater stigma and insufficiency of self-identification, which are associated with poor mental and physical health, including nonsuicidal self-injury and suicidal behaviours (Galan *et al*., 2021). In addition, interestingly, adolescents who were left out of activities on purpose or completely ignored had the highest probability of suicide planning, although this type of bullying had the lowest incidence. These results remind us that focusing on the types of bullying that are more likely to lead to suicidal behaviour may be an effective intervention and that the serious psychological impact of relational bullying on teenagers cannot be ignored.

Furthermore, consistent with hypothesis 3, the associations between bullying victimisation and suicidal behaviours varied in different groups of teenagers. The effects of bullying victimisation were stronger on males' suicidal behaviours. A possible explanation of our result is that males, in general, are socially expected to exhibit self-esteem and success (Falah and Ibrahim, 2017), which are more sensitive to being bullied. Moreover, adolescents who were underweight and overweight or obese had significantly greater odds than their healthy-weight peers of suicidal behaviours after being victims of bullying, and the effects were strongest among obese adolescents. The mechanism of this regulation is still unclear. We consider that this finding may be related to the fact that these adolescents are more likely to be bullied (Rupp and McCoy, 2019) and have more emotion regulation difficulties and less interoceptive awareness and are more likely to have suicidal impulses (Willem *et al*., 2019). Based on these results, male, overweight and obese adolescents may need urgent attention. Efforts to reduce bullying, especially at school, may be fundamental to prevent or reduce adolescent suicides.

### Limitations and implications

Several limitations of this work should be considered. First, the GSHS's cross-sectional design precludes temporal and causal inference, and future longitudinal studies are needed to provide more insight into causality. Second, the assessments in this research were based on single questions without structured instruments, rendering the study much more prone to measurement error. Third, this study did not assess the comorbidity among the types of bullying due to the nature of the questions (which were not multiple choice). Despite these limitations, the current study has important theoretical and practical implications (provided in the online Supplementary materials).

Overall, this study provides important insight into the associations between different types of bullying victimisation and suicidal behaviours. Offering a theoretical basis to construct a sound school environment would improve adolescents' mental health and reduce the risk of suicide to promote global health. From a practical perspective, our findings may inform the design of effective psychological interventions to reduce the incidence of teenage suicide. Future longitudinal studies are needed to provide more insight into causality and the potential mediators (e.g. depression) or moderators (e.g. parental support) that are involved in the association of bullying victimisation and suicidal behaviours.

## Conclusions

In summary, the prevalence of bullying victimisation and suicidal behaviours are high among school adolescents in LMICs. Those who are bullied have higher odds of suicidal behaviours than those who are not bullied, and being made fun of because of religion has the strongest effect. Moreover, these associations vary in teenagers of different genders and BMIs. Thus, school bullying is a silent public health concern that requires a dedicated team of families, educators, healthcare professionals and policy-makers to mitigate. Longitudinal studies are needed to provide more insight into causality, and subsequent studies should aim to ascertain the role of conditioning variables that mediate and moderate the relationship between bullying and suicidal behaviours for the establishment of effective interventions to counteract this global problem.

## Data Availability

We used the most recent Global School-based Student Health Survey (GSHS) data (2010–2017) from 40 LMICs or regions, which are presented on the websites of the WHO (http://www.who.int/chp/gshs) and the U.S. Centers for Disease Control and Prevention (CDC) (http://www.cdc.gov/gshs).

## Supplementary material

For supplementary material accompanying this paper visit http://doi.org/10.1017/S2045796022000440. click here to view supplementary material
