# Supplementary material for: Associations of bullying victimisation in different frequencies and types with suicidal behaviours among school-going adolescents in low- and middle-income countries
Source: Epidemiol Psychiatr Sci. 2022 Aug 11;31:e58. doi: 10.1017/S2045796022000440 (PMC9387118; doi:10.1017/S2045796022000440)
Supplement: Supplementary file 1 [file epssup.zip › S2045796022000440sup001.docx]

Online Supplementary Material 1: Detailed information about the survey.

***The survey (GSHS)***

The core purpose of the GSHS was to help countries develop priorities, establish programs, and advocate for resources for school health and youth health programs and policies. In this study, publicly available data from the GSHS (2010–2017) conducted in 40 LMICs or regions (from the African, Americas, Eastern Mediterranean, Southeast Asia, and Western Pacific regions) were analysed, and only the most recent data from each country were used.

The survey used a standardized two‐stage probability sampling design for the selection process within each participating country. In each participating country, in the first stage, researchers randomly selected schools from a country using the probability proportionate to size method. In the second stage, systematic equal probability sampling with random start was used to select classes from the schools to include in the survey. All of the students in the selected school classes were included in the sampling frame. The GSHS questionnaire comprised ten modules of questions on health, such as mental health and protective factors. Various modules could be selected for a given country, but the questions from the selected modules were not modifiable. The questionnaire could be translated into any language, as appropriate. Notably, all responses were anonymous, and no personal identifiable information was collected. The GSHS used an automated optic character recognition procedure to identify students’ records on computer-scannable answer sheets and to collect data.

The GSHS administration was approved by the Ministry of Education, a health research ethics committee, or both. Verbal or written consent was obtained from all the participants and their guardians in each country. The present study did not require the approval of ethics or institutional review boards because the analyses are based on publicly available data.
