# Supplementary material for: Associations of bullying victimisation in different frequencies and types with suicidal behaviours among school-going adolescents in low- and middle-income countries
Source: Epidemiol Psychiatr Sci. 2022 Aug 11;31:e58. doi: 10.1017/S2045796022000440 (PMC9387118; doi:10.1017/S2045796022000440)
Supplement: Supplementary file 1 [file epssup.zip › S2045796022000440sup002.docx]

# Online Supplementary Material 2: Detailed Description of Results

***Differences in bullying victimization and suicidal behaviours***

Table 2 and its supplement show the difference in bullying victimization during the past 30 days as follows: a). Age: The prevalence of bullying victimization among students aged 11-13, 14, 15, 16, and 17-18 years or older was 24.82%, 23.91%, 22.58%, 21.21% and 19.04%, respectively. Being made fun of because of how one’s body or face looks had the highest prevalence among students aged 11-13, 14, 15, and 16 years old at 3.99%, 4.32%, 4.08% and 3.85%, respectively. In students aged 17-18 years old or older, being made fun of with sexual jokes, comments or gestures (2.97%) had the highest prevalence. b). Gender: The prevalence of bullying victimization among male students was 23.6%, and there was a higher prevalence of being made fun of with sexual jokes, comments or gestures (4.15%) and being hit, kicked, pushed, shoved around, or locked indoors (4.12%) among male students than among female students. The prevalence of bullying victimization among female students was 22.04%, and there was a higher prevalence of being made fun of because of how one’s body or face looked (4.52%) and being made fun of with sexual jokes, comments or gestures (3.48%) among female students than among male students. c). BMI: Students with BMIs lower than 18.5 had the highest prevalence of bullying victimization. The prevalence of being made fun of with sexual jokes, comments or gestures was highest in students with BMI lower than 18.5 (4.37%) and from 18.5 to 24.9 (3.65%), while students with BMI from 25 to 30 (5.63%) and over 30 (8.11%) had the highest prevalence of being made fun because of how one’s body or face looks. d). Geographic areas: The prevalence of bullying victimization was highest in students from Africa (30.41%) and lowest in the Eastern Mediterranean (10.68%). These areas both had the highest prevalence of being hit, kicked, pushed, shoved around, or locked indoors, at 5.75% and 2.17%, respectively. Being made fun of because of how one’s body or face looks had the highest prevalence among students from the Americas (4.87%). Being made fun of with sexual jokes, comments or gestures had the highest prevalence in students from Southeast Asia (4.56%) and the Western Pacific (4.46%). e). Other control variables: The prevalence of bullying victimization was higher among students with food insecurity (27.58%), smoking (30.14%), alcohol use (28.27%), marijuana use (35.07%), school absence (28.38%), no parental understanding (22.93%), no close friends (27.28%), loneliness (27.7%) and sleeping difficulties (27.98%) than among their counterparts. In addition, these students all experienced a higher prevalence of being made fun of about sex, being made fun of because of how one’s body or face looks and being hit, kicked, pushed, shoved around, or locked indoors.

The analysis of the interaction between area and age shows that the risk of being made fun of with sexual jokes, comments or gestures was the highest among the 17- to 18-year-old students from Africa (OR = 1.39, 95% CI = 1.16-1.66) and the Americas (OR = 1.32, 95% CI = 1.01-1.72), 16-year-old students from Southeast Asia (OR = 1.89, 95% CI = 1.57-2.28) and 14-year-old students from the Western Pacific region (OR = 2.02, 95% CI = 1.77-2.30). The risk of being made fun of because of how one’s body or face looks was the highest among the 11- to 13-year-old students from Southeast Asia (OR = 1.31, 95% CI = 1.10-1.56), 14-year-old students from the Americas (OR = 2.23, 95% CI = 1.93-2.58) and Western Pacific (OR = 1.87, 95% CI = 1.62-2.16), and 16-year-old students from Africa (OR = 2.54, 95% CI = 2.08-3.11). The analysis of the interaction between area and gender shows that the risk of being kicked, pushed, or shoved (OR = 3.11, 95% CI = 2.79-3.46) was the highest among males in Africa, the risk of being made fun of with sexual jokes, comments or gestures (5.7%) was the highest among male students from Southeast Asia, and the risk of being made fun of because of how one’s body or face looks was the highest among African (OR = 1.28, 95% CI = 1.15-1.43) and American (OR = 1.47, 95% CI = 1.33-1.62) females.

In addition, Table 3 and its supplement show the difference in suicidal behaviours in the same aspects. a). Age: The prevalence of suicidal behaviours decreased with increasing age. Students aged 11-13 years old had the lowest prevalence of suicidal ideation (10.64%), suicidal plans (9.95%) and suicidal attempts (9.09%), and students aged 17-18 years old or older had the highest prevalence, which were 13.12%, 13.21% and 11.2%, respectively. b). Gender: Female students had a higher prevalence of suicidal ideation (15.01%), suicidal plans (13.51%) and suicidal attempts (12.15%). c). BMI: The prevalence of suicidal ideation (10.11%), suicidal plans (9.46%) and suicid attempts (8.85%) in students with BMIs lower than 18.5 was significantly lower than that in the other groups. d). Areas: Students in the Americas (17.43%) had the highest prevalence of suicidal ideation, and the prevalence of suicidal plans (18.18%) and suicidal attempts (16.55%) were highest in Africa. Students in Southeast Asia had the lowest prevalence of suicidal behaviours, at 7.72% (suicidal ideation), 7.84% (suicide plan), and 5.18% (suicidal attempts). e). Other control variables: The prevalence of suicidal ideation was higher in students with food insecurity (14.53%), smoking (24.48%), alcohol use (21.58%), marijuana use (31.79%), school absence (17.00%), no parental understanding (18.38%), no close friends (22.08%), loneliness (15.29%) and sleeping difficulties (16.14%) than in their counterparts. The prevalence of suicidal plans and suicidal attempts were the same, and details are shown in the table.

***Associations between bullying victimization and suicidal behaviours***

In the multinomial logistic regression models (Table 4), the associations between bullying victimization and suicidal behaviour were significant after controlling for age, gender, BMI, area, food insecurity, delinquent conduct, other mental health problems, and respondents’ experiences at home and school. a). Frequency of bullying victimization: Taking 0 days of bullying victimization as a reference, the risk of suicidal ideation at 1 or 2 days (OR=1.30, 95% CI=1.22-1.39), 3 to 5 days (OR=1.53, 95% CI=1.40-1.67), 6 to 9 days (OR=1.83, 95% CI=1.64-2.04), 10 to 19 days (OR=2.07, 95% CI=1.82-2.35), 20 to 29 days (OR=2.43, 95% CI=2.06-2.87) and all 30 days (OR=2.31, 95% CI=2.07-2.56) increased. The odds of suicidal plans and suicidal attempts also increased with increasing frequency of bullying victimization; details are shown in the table. b). Different types of bullying: The associations of being made fun of because of religion (OR=1.63, 95% CI=1.41-1.88), being left out of activities on purpose or completely ignored (OR=1.48, 95% CI=1.31-1.68), being hit, kicked, pushed, shoved around, or locked indoors (OR=1.47, 95% CI=1.33-1.62), being made fun of with sexual jokes, comments or gestures (OR=1.46, 95% CI=1.33-1.60) and being made fun of because of how one’s body or face looks (OR=1.45, 95% CI=1.32-1.58) with suicidal ideation were strongest. For suicidal plans, being made fun of because of religion (OR=1.44, 95% CI=1.24-1.66) and being hit, kicked, pushed, shoved around, or locked indoors (OR=1.44, 95% CI=1.31-1.59) had the strongest associations. Being made fun of because of religion (OR=1.73, 95% CI=1.50-1.98) also had the strongest relation to suicidal attempts, followed by being hit, kicked, pushed, shoved around, or locked indoors (OR=1.41, 95% CI=1.29-1.55).

Furthermore, the results of stratified logistic regressions conducted according to participants’ gender and BMI are shown in Figure 2. a). Gender: Compared to females, males experiencing bullying victimization had a higher risk of suicidal ideation (OR=1.17, 95% CI=1.15-1.20), suicidal plans (OR=1.17, 95% CI=1.14-1.19) and suicidal attempts (OR=1.18, 95% CI=1.16-1.21). b). BMI: Students with a BMI of 18.5 to 24.9 had the lowest risk of suicidal plans (OR=1.13, 95% CI=1.10-1.15) and suicidal attempts (OR=1.16, 95% CI=1.14-1.18), and students with a BMI of 25 to 30 had the lowest risk of suicidal ideation (OR=1.13, 95% CI=1.08-1.19). Meanwhile, the risk of suicidal ideation (OR=1.28, 95% CI=1.19-1.37) and suicidal attempts (OR=1.21, 95% CI=1.12-1.31) was highest in students with a BMI greater than 30, and the risk of suicidal plans (OR=1.19, 95% CI=1.15-1.23) was highest in students with a BMI lower than 18.5.
