# Supplementary material for: Associations of bullying victimisation in different frequencies and types with suicidal behaviours among school-going adolescents in low- and middle-income countries
Source: Epidemiol Psychiatr Sci. 2022 Aug 11;31:e58. doi: 10.1017/S2045796022000440 (PMC9387118; doi:10.1017/S2045796022000440)
Supplement: Supplementary file 1 [file epssup.zip › S2045796022000440sup003.docx]

# Online Supplementary Material 3: Tables

| **Supplementary Table S1. Sample Characteristics. (*n* = 151,184)** | | |
| --- | --- | --- |
|  | ***n*** | **%** |
| Area |  |  |
| African Region | 17,802 | 11.78 |
| Region of the Americas | 37,470 | 24.78 |
| Eastern Mediterranean Region | 4,476 | 2.96 |
| South-East Asia Region | 24,623 | 16.29 |
| Western Pacific Region | 66,813 | 44.19 |
| Food insecurity |  |  |
| No | 73,919 | 48.89 |
| Yes | 77,265 | 51.11 |
| Smoking cigarette |  |  |
| No | 133,352 | 88.21 |
| Yes | 17,832 | 11.79 |
| Alcohol use |  |  |
| No | 118,654 | 78.48 |
| Yes | 32,530 | 21.52 |
| Marijuana use |  |  |
| No | 146,063 | 96.61 |
| Yes | 5,121 | 3.39 |
| Missed school |  |  |
| No | 108,835 | 71.99 |
| Yes | 42,349 | 28.01 |
| Parental understanding |  |  |
| No | 33,437 | 22.12 |
| Yes | 117,747 | 77.88 |
| Have close friends |  |  |
| No | 7,750 | 5.13 |
| Yes | 143,434 | 94.87 |
| Loneliness |  |  |
| No | 51,347 | 33.96 |
| Yes | 99,837 | 66.04 |
| Sleeping difficulty | | |
| No | 57,704 | 38.17 |
| Yes | 93,480 | 61.83 |

| **Supplementary Table S2. Factors associated with bullying victimizations among adolescents. (*n* = 151,184)** | | | | | | | | |  |
| --- | --- | --- | --- | --- | --- | --- | --- | --- | --- |
|  | **Different types of bullying (%)** | | | | | | | | Rao-Scott Chi-Square *p* |
|  | Not bullied | Type 1 | Type 2 | Type 3 | Type 4 | Type 5 | Type 6 | Type 7 |  |
| Food insecurity |  |  |  |  |  |  |  |  | <0.001 |
| No | 82.29 | 2.29 | 1.60 | 0.63 | 2.61 | 1.04 | 3.06 | 6.47 |  |
| Yes | 72.42 | 3.85 | 3.28 | 1.23 | 4.91 | 1.74 | 4.71 | 7.85 |  |
| Smoking cigarette |  |  |  |  |  |  |  |  | <0.001 |
| No | 78.24 | 2.84 | 2.37 | 0.89 | 3.53 | 1.39 | 3.81 | 6.94 |  |
| Yes | 69.86 | 4.97 | 3.15 | 1.31 | 5.73 | 1.49 | 4.59 | 8.91 |  |
| Alcohol use |  |  |  |  |  |  |  |  | <0.001 |
| No | 78.76 | 2.90 | 2.33 | 0.89 | 3.44 | 1.36 | 3.61 | 78.76 |  |
| Yes | 71.73 | 3.79 | 2.94 | 1.11 | 5.05 | 1.55 | 4.96 | 71.73 |  |
| Marijuana use |  |  |  |  |  |  |  |  | <0.001 |
| No | 77.68 | 2.96 | 2.36 | 0.88 | 3.75 | 1.38 | 3.87 | 77.68 |  |
| Yes | 64.93 | 6.91 | 5.31 | 2.44 | 4.92 | 1.84 | 4.73 | 64.93 |  |
| Missed school |  |  |  |  |  |  |  |  | <0.001 |
| No | 79.44 | 2.56 | 2.16 | 0.77 | 3.40 | 1.30 | 3.64 | 6.73 |  |
| Yes | 71.62 | 4.44 | 3.23 | 1.37 | 4.80 | 1.65 | 4.59 | 8.31 |  |
| Parental understanding |  |  |  |  |  |  |  |  | <0.001 |
| No | 77.86 | 3.42 | 2.55 | 0.92 | 3.62 | 1.36 | 4.00 | 6.28 |  |
| Yes | 77.07 | 3.00 | 2.44 | 0.94 | 3.84 | 1.41 | 3.87 | 7.43 |  |
| Have close friend |  |  |  |  |  |  |  |  | <0.001 |
| No | 72.72 | 4.70 | 3.73 | 1.45 | 3.43 | 2.12 | 4.67 | 7.19 |  |
| Yes | 77.49 | 3.00 | 2.39 | 0.91 | 3.81 | 1.36 | 3.86 | 7.18 |  |
| Loneliness |  |  |  |  |  |  |  |  | <0.001 |
| No | 86.87 | 2.15 | 1.39 | 0.71 | 1.66 | 0.51 | 1.86 | 4.85 |  |
| Yes | 72.30 | 3.57 | 3.01 | 1.05 | 4.88 | 1.86 | 4.95 | 8.37 |  |
| Sleeping difficulty |  |  |  |  |  |  |  |  | <0.001 |
| No | 85.72 | 2.26 | 1.54 | 0.62 | 2.09 | 0.74 | 2.17 | 4.86 |  |
| Yes | 72.02 | 3.60 | 3.03 | 1.13 | 4.84 | 1.80 | 4.98 | 8.61 |  |

*Note:* Type 1: Kicked, pushed, or shoved around, or locked indoors; Type 2: Made fun of race, nationality, or colour; Type 3: Made fun because of religion; Type 4: Made fun of with sexual jokes, comments or gestures; Type 5: Left out of activities on purpose or completely ignored; Type 6: Made fun of about body or face looks; Type 7: Some other way.

| **Supplementary Table S3. Factors associated with suicidal behaviours among adolescents. (*n* = 151,184)** | | | | | | | |
| --- | --- | --- | --- | --- | --- | --- | --- |
|  | **Suicidal ideation** | | **Suicidal plans** | | **Suicidal attempts** | | |
|  | prevalence (%) | *p* | Prevalence (%) | *p* | | Prevalence (%) | *p* |
| Have close friends |  | <0.001 |  | <0.001 | |  | <0.001 |
| No | 22.08 |  | 23.64 |  | | 21.59 |  |
| Yes | 12.14 |  | 11.20 |  | | 10.20 |  |
| Loneliness |  | <0.001 |  | <0.001 | |  | <0.001 |
| No | 7.51 |  | 7.99 |  | | 6.64 |  |
| Yes | 15.29 |  | 13.81 |  | | 12.92 |  |
| Sleeping difficulty |  | <0.001 |  | <0.001 | |  | <0.001 |
| No | 6.98 |  | 7.37 |  | | 5.93 |  |
| Yes | 16.14 |  | 14.59 |  | | 13.78 |  |
